# ‘Now I Have Dreams in Place of the Nightmares’: An Updated Systematic Review of Post-Traumatic Growth Among Refugee Populations

**DOI:** 10.1177/15248380231163641

**Published:** 2023-04-25

**Authors:** Grace Sultani, Milena Heinsch, Jessica Wilson, Phillip Pallas, Campbell Tickner, Frances Kay-Lambkin

**Affiliations:** 1 University of Newcastle, Callaghan, Australia

**Keywords:** post-traumatic growth, refugees, trauma, positive transformation, coping

## Abstract

Trauma exposure places refugees at serious risk of developing mental health difficulties. However, research also recognizes that refugees can respond to trauma with psychological development and growth, commonly referred to as post-traumatic growth (PTG). An updated systematic review was conducted to investigate PTG across different refugee populations, including the processes that mediate this phenomenon, and the use of therapy in promoting PTG. A systematic search of CINAHL Complete, Proquest 5000, PsychINFO, Scopus, and Web of Science was performed to identify studies exploring PTG in refugee populations, published between June 2013 and November 2021. In all, 26 studies met the inclusion criteria for this review. Quantitative results reveal a positive correlation between PTG and religious commitment and coping, and the effectiveness of narrative and community-based interventions in facilitating PTG. Qualitative results facilitate insight into the complex ways refugees find meaning and strength after trauma through religion, comparison-based thinking, helping others, and storytelling. Findings highlight the need for future research and interventions to recognize the distinct PTG experiences of different refugee populations.

## Introduction

Worldwide, 100 million people have been forcibly displaced from their homes after being exposed to war, conflict, persecution, or natural disaster (United Nations High Commissioner for Refugees, n.d.-a). Refugee experiences often include significant emotional distress, resulting from the denial and abuse of their basic human rights (NSW Servive for the Treatment and Rehabilitation of Torture and Trauma Survivors, n.d). Consequently, refugees are at serious risk of developing mental health issues, including post-traumatic stress disorder (PTSD), anxiety, and depression (Gojer & Ellis, 2014; Sleijpen et al., 2013). While it is important that these detrimental outcomes are documented in the literature, also important is acknowledging that not all individuals exposed to trauma will experience negative mental health outcomes (Kira et al., 2013). Papadopoulos (2007) warned that excessive pathologizing of refugee suffering risks overlooking the complex and wide-ranging psychological experiences humans can have following trauma, and the positive outcomes that can ensue from an individual’s response to these encounters.

The human capacity for positive transformation following adversity is not a new concept in the wider literature (Chiovenda, 2021). The teachings and traditions of several religions, ranging from Christianity, Hinduism, Buddhism, and Islam, contain narratives of the potential for transformation after suffering (Tedeschi & Calhoun, 2004a). Notable early accounts of psychological transformation after the struggle with trauma also include Viktor Frankl’s (1946) autobiography of survival and growth in World War II concentration camps, and Carl Jung’s examination of how positive aspects of psychological distress can assist in the establishment of a balanced psyche (Chiovenda, 2021). Gerald Caplan, too, extensively explored the potential for psychological development after adversity as a result of effective coping (Tedeschi & Calhoun, 2004a). Over the past three decades, literature exploring personal development as a result of an individual’s response to adversity has employed a variety of terms, including “stress-related growth” (Park et al., 1996), “benefit finding” (Affleck & Tennen, 1996), “positive psychological change” (Yalom & Lieberman, 1991), “transformation of trauma” (Calhoun & Tedeschi, 1989), “adversarial growth” (Linley & Joseph, 2004), and “perceived benefits” (McMillen et al., 1997). In recent years, Tedeschi and Calhoun’s (1995) “post-traumatic growth” (PTG) has emerged as the dominant concept in the literature (Papadopoulos, 2007).

PTG suggests that individuals can experience growth in diverse areas of their life through the cognitive processing of trauma, potentially improving their functioning beyond baseline levels (Tedeschi & Calhoun, 2004a). According to Tedeschi and Calhoun (1996), growth occurs in five key domains: (1) relating to others, (2) appreciation of life, (3) personal strength, (4) new possibilities, and (5) spiritual change (see Table 1 for a brief description of these domains). Tedeschi and Calhoun (2004a) caution that growth does not occur as a result of trauma; rather, as the result of an individual’s “struggle with highly challenging life circumstances” in the aftermath of trauma (p. 1). Interestingly, the paradox of PTG is that positive change does not imply an absence of distress or negative implications from trauma (Chiovenda, 2021). Instead, an individual can simultaneously be “more vulnerable” and “yet stronger,” demonstrating that loss can result in important gains (Tedeschi & Calhoun, 2004b, p. 2).

**Table 1.** Description of PTG Domains.

PTG Domain	Description
Relating to others	Development of more intimate and meaningful relationships, often accompanied by an increase in compassion toward others
Appreciation of life	Increased appreciation of life, change in priorities, and recognizing the importance of things previously taken for granted
Personal strength	Increased recognition of one’s strength, and ability to survive and manage hardship, often resulting in a greater sense of self-reliance
New possibilities	Recognizing the availability and possibility of new, or different pathways in life, and a willingness to explore these opportunities
Spiritual change	A deeper level of spiritual and/or religious beliefs or awareness, and/or increased engagement with existential thoughts

*Source.* Tedeschi and Calhoun (2004a).

PTG has been used to explore a variety of traumatic experiences, including loss and bereavement (Büchi et al., 2007), sexual assault (Lev-Wiesel et al., 2005), domestic violence (Counselman-Carpenter & Redcay, 2018), natural disasters (Cryder et al., 2006), health and medical issues (Cordova et al., 2001), and refugee experiences (Powell et al., 2003). The positive changes experienced by refugees as a result of their struggle with trauma have been found to align with Tedeschi and Calhoun’s (1996) five domains, suggesting that PTG is a relevant framework for exploring the ways in which refugees can respond to adversity. While other theoretical frameworks explore positive coping and protective factors, PTG is unique in its focus on transformation after trauma, and the domains in which this transformation occurs.

While PTG research with refugees has increased in recent years, to our knowledge, only three reviews have synthesized the empirical literature in this area. In 2016, Chan et al. conducted a literature review investigating key factors facilitating positive transformation among refugees. In 2017, Sims and Pooley (2017) conducted a systematic review exploring PTG across different refugee populations, including the factors that mediate growth and the use of therapy in promoting PTG among different refugee populations. Following publication of these reviews, a substantial number of studies have investigated the relationship between PTG and post-traumatic stress (Acquaye, 2017; Cengiz et al., 2019; Ersahin, 2020; Rizkalla & Segal, 2018; Simsir & Dilmac, 2021), the effectiveness of interventions to promote PTG (Hijazi et al., 2014; Paloma et al., 2019), and the impact of resettlement experiences on PTG responses (Acar et al., 2021; Alsubaie et al., 2021; Özdemir et al., 2021; Prasetya et al., 2020; Rizkalla & Segal, 2018). These studies correspond with areas for future research specifically identified by Chan et al. (2016) and Sims and Pooley (2017), highlighting the need for an updated synthesis that considers the implications of these recent research findings for researchers and practitioners who work with refugees.

Recently, Şimşir Gökalp and Haktanir (2022) conducted a metasynthesis of qualitative studies exploring the PTG experiences of refugees. While this review provided important insights into the PTG experiences of different cultural groups, a sole focus on qualitative studies risks overlooking the understandings that can be gained from quantitative measures like the Post-Traumatic Growth Inventory (PTGI), a 21-item measure that uses a six-point Likert scale to determine perceived levels of growth against the five key domains of PTG (Tedeschi & Calhoun, 1996). For example, a comparison of PTGI mean scores may enable identification of different levels of PTG among refugee populations (Sims & Pooley, 2017), or changes in PTG following intervention. Thus, to enable consideration of both qualitative and quantitative findings, we updated Sims and Pooley’s (2017) systematic review to investigate the most recent empirical PTG literature across refugee populations, including the processes that mediate this phenomenon, and the use of therapy in promoting PTG among refugees. Garner et al. (2016) note that when addressing the same research question, an updated review is more efficient than a new protocol as it enables improvement in the search strategy and methods over time. Findings from this review are discussed with reference to Tedeschi and Calhoun’s (1996) five domains of PTG, highlighting important new directions for research and practice with this population.

## Method

This updated systematic review was informed by the Preferred Reporting Items for Systematic Reviews and Meta-Analyses (PRISMA) guidelines (Liberati et al., 2009; Page et al., 2021). The authors adopted a flexible approach; continuing to apply the core principles of systematic review methodology, while tailoring the PRISMA guidelines to the needs of this review (Mallett et al., 2012).

### Search Strategy

The research questions guiding this review were:

1. What is known about PTG across different refugee populations?
2. What is known about the processes that mediate PTG among refugee populations?
3. What is known about the use of therapy in promoting PTG among refugee populations?

Our search strategy followed that of Sims and Pooley (2017), with key search terms including: (“post-traumatic growth” or “PTG” or “posttraumatic growth” or “positive change” or “benefit finding” or “stress-related growth” or adversarial growth” or “positive psychological change” or “perceived benefits” or “transformational coping”) AND (“refugee” or “asylum seeker” or “humanitarian entrant” or “displaced person” or “forced migrant”) AND (“meditate” or “moderate”) AND (“therapy” or “therapeutic intervention” or “psychological intervention” or “intervention” or “clinical intervention” or “clinical practice” or “mental health care”) AND (“promoting” or “facilitate” or “support” or “help” or “encourage” or “advance” or “further” or “nurture” or “assist” or “enable” or “aid” or “stimulate”). The search was conducted in the databases of CINAHL Complete, Proquest 5000, PsychINFO, Scopus, and Web of Science.

### Eligibility Criteria

Articles included in the review were as follows: (1) published between June 2013 and November 2021 (following on from Sims and Pooley’s (2017) review, which included articles published prior to June 2013); (2) peer-reviewed empirical studies (qualitative, quantitative, or mixed methods); (3) dissertations/theses; (4) published in English; and (5) examined PTG in adult refugees, asylum seekers, and/or internally displaced people (IDPs) >18 years of age. Excluded articles were as follows: (1) published prior to June 2013; (2) published in a language other than English; (3) grey literature not published in a peer-reviewed journal; and (4) explored PTG in children or young people <18 years of age. Studies were excluded if they did not state the age of participants. Studies involving former refugees were included, in recognition that refugee experiences continue to stay with a person after resettlement occurs (McCormack & Strezov, 2021).

### Identification and Inclusion of Studies

Identified articles were screened by two reviewers (GS and CT) using the web-based software Covidence (Veritas Health Innovation Ltd, Melbourne, Victoria, Australia). Reviewers independently conducted title, abstract, and full-text screening of all potentially eligible papers. Any discrepancies in the exclusion of papers were discussed between the two reviewers or, if needed, with senior author (MH). Reference lists of eligible articles were manually scanned for any papers missed during the database search.

### Data Extraction and Presentation

Table 2 summarizes the key characteristics of included articles: (i) author and date, (ii) sample total and gender breakdown, (iii) research design, (iv) country of origin, (v) religion, (vi) host country or resettlement country, (vii) time elapsed since relocation, (viii) measure of PTG, (ix) PTGI mean scores (when specified), and (x) standard deviation. Quantitative results are presented first, followed by a thematic synthesis of qualitative findings (Thomas & Harden, 2008), reported in narrative form for a more complete and nuanced story (Popay et al., 2006) that foregrounds the voices of refugee participants (Saltsman & Majidi, 2021). Throughout this review, the term “refugees” is used as an umbrella term that includes asylum seekers, former refugees, and internally displaced persons.

**Table 2.** Summary of the Key Characteristics of Included Studies.

Author, Date	Sample Total, Gender Breakdown	Research Design	Country of Origin	Religion	Host Country or Resettlement Country	Time Elapsed Since Relocation	Measure of PTG	PTGI Mean Scores	Standard Deviation
Abraham et al. (2018)	18 participants, 100% female	Qualitative focus groups and interviews	Eritrea	Not stated	Norway	1–8 years			
Acar et al. (2021)	528 participants, (52% female, 47.1% male)	Cross-sectional survey	Syria	Not stated	Turkey	Average 5.5 years	PTGI Arabic version		
Acquaye (2017)	444 participants (29.1% female, 70.9% male)	Cross-sectional survey	Liberia	Not stated	Liberia	Not stated	PTGI	84.49 (Female)	16.03 (Female)
								79.56 (Male)	17.66 (Male)
Acquaye et al. (2018)	444 participants (29.1% female, 70.9% male)	Cross-sectional survey	Liberia	Christian, Muslim	Liberia	Not stated	PTGI		
Alsubaie et al. (2021)	112 participants (28.6% female, 71.4% male)	Cross-sectional survey	Multiple countries of origin	Muslim	Multiple countries of resettlement	Not stated	PTGI-SF	28.86	9.74
Cengiz et al. (2019)	310 participants (47.1% female, 52.9% male)	Cross-sectional survey	Syria	Not stated	Turkey	1 to 3+ years	PTGI Arabic version	62.54 (PTSD)	18.54 (PTSD)
								56.68 (Non-PTSD)	20.90 (Non-PTSD)
Dolezal et al. (2021)	112 participants (28.6% female, 71.4% male)	Cross-sectional survey	Multiple countries of origin	Muslim	Multiple countries of resettlement	Not stated	PTGI-SF	28.86 (Full sample)	9.75 (Full sample)
								31.24 (Refugees)	8.73 (Refugees)
								25.33 (Asylum seekers)	10.61 (Asylum seekers)
								29.04 (IDPs)	9.25 (IDPs)
Ersahin (2020)	805 participants (47.5% female, 40.8% male, 11.5% no response)	Cross-sectional survey	Syria	Muslim, Christian, Jewish, no religion, other	Turkey	Average 6.17 years	PTGI Arabic version	54.17 (Female)	23.63 (Female)
								51.15 (Male)	29.96 (Male)
Ferriss and Forrest-Bank (2018)	12 participants (50% female, 50% male)	Qualitative focus groups	Somalia	Muslim	USA	1.3–15 years			
Hijazi et al. (2014)	63 participants (55.6% female, 44.4% male)	Randomized clinical trial	Iraq	Chaldean	USA	Average 2.3 years	PTGI	45.78 (NET baseline)	23.85 (NET baseline)
								51.22 (NET 2 months)	25.40 (NET 2 months)
								58.18 (NET 4 months)	22.44 (NET 4 months)
								47.68 (Non-NET baseline)	22.66 (Non-NET baseline)
								41.86 (Non-NET 2 months)	21.73 (Non-NET 2 months)
								39.58 (Non-NET 4 months)	39.58 (Non-NET 4 months)
Hirad (2017)	30 participants (43.3% female, 56.6% male)	Qualitative interviews	Iraq, Syria, Afghanistan, and Iran	Muslim, Christian, Zoroastrian, Mormon	USA	Less than 1 year to more than 10 years			
Matos et al. (2021)	39 participants (48.7% female, 51.3% male)	Qualitative semi-structured cognitive interviews	Syria	Various religions	Portugal	11 months to 5.5 years			
Maung et al. (2021)	11 participants (100% female)	Qualitative, semi-structured interviews	Burma	Christian, Muslim	USA	3–11 years			
McCormack and Strezov (2021)	5 participants (60% female, 40% male)	Qualitative semi-structured interviews	Africa, Macedonia, Tibet, and Poland	Not stated	Australia and Macedonia	Not stated			
Özdemir et al. (2021)	72 participants (46% female, 54% male)	Cross-sectional survey	Syria	Not stated	Turkey	Not stated	PTGI	62.30	18.25
Paloma et al. (2019)	47 participants (43% female, 57% male)	Longitudinal, mixed methods, using the PTGI and written assessments	Multiple countries of origin	Not stated	Spain	Not stated	PTGI Spanish version	78.67 (Pre-intervention)	12.83 (Pre-intervention)
								89 (Post-intervention)	10.07 (Post-intervention)
Prasetya et al. (2020)	97 participants (53.6% female, 46.4% male)	Cross-sectional survey	Indonesia	Not stated	Indonesia	Average 6.5 years			
Rizkalla and Segal (2018)	250 participants (54.6% female, 45.4% male)	Cross-sectional survey	Syria	Muslim, other	Jordan	36 months and less	PTGI	51.36	19.90
Shakespeare-Finch et al. (2014)	25 participants (52% female, 48% male)	Qualitative interviews	Burma	Christian, Buddhist, Muslim, no religion	Australia	12 months and less			
Simsir et al. (2021)	15 participants (66.6% female, 33.3% male)	Qualitative semi-structured interviews	Syria	Muslim	Turkey	1–4 years			
Simsir and Dilmac (2021)	303 participants (65.3% female, 34.7% male)	Cross-sectional survey	Syria	Muslim	Turkey	Not stated	PTGI		
Taher and Allan (2020)	54 participants (42.6% female, 57.4% male)	Mixed methods using the PTGI and semi-structured interviews	Syria	Not stated	UK	Not stated	PTGI	70.74	17.55
Taylor et al. (2020)	12 participants (75% female, 25% male)	Qualitative, two-stage interview process	Multiple countries of origin	Not stated	UK	5–21 years			
Umer and Elliot (2021)	16 participants (37.5% female, 62.5% male)	Mixed methods, using the PTGI and narrative writing task	Syria, Palestine, Sudan	Not stated	UK	Less than 1 year	PTGI		
Uy and Okubo (2018)	12 participants (33.3% female, 66.6% male)	Qualitative, semi-structured interviews	Cambodia	Buddhist, not stated	USA	31–47 years			
Wen et al. (2020)	767 participants (40.7% female, 59.2% male)	Cross-sectional survey	Syria	Not stated	Turkey	Not stated	PTGI	55.94	**22.91**

IDP = internally displaced people; NET = narrative exposure therapy; PTGI = post-traumatic growth inventory; PTSD = post-traumatic stress disorder.

### Quality Assessment

The Quality Assessment for Diverse Studies (QuADS) tool was used to assess the methodological rigor of the studies included in this review (Harrison et al., 2021). The tool includes 13 items assessing background and theoretical approach, transparency of aims and research methods, and overall appropriateness of study design and analysis. Each item is rated on a 0–3 scale, with the total possible score ranging from 0 to 39. The tool shows good inter-rater reliability (*K* = .66) and strong content and face validity (Harrison et al., 2021). Two reviewers (GS and JW) independently completed the quality assessment of all included articles.

## Results

### Search Results

**Figure 1.**
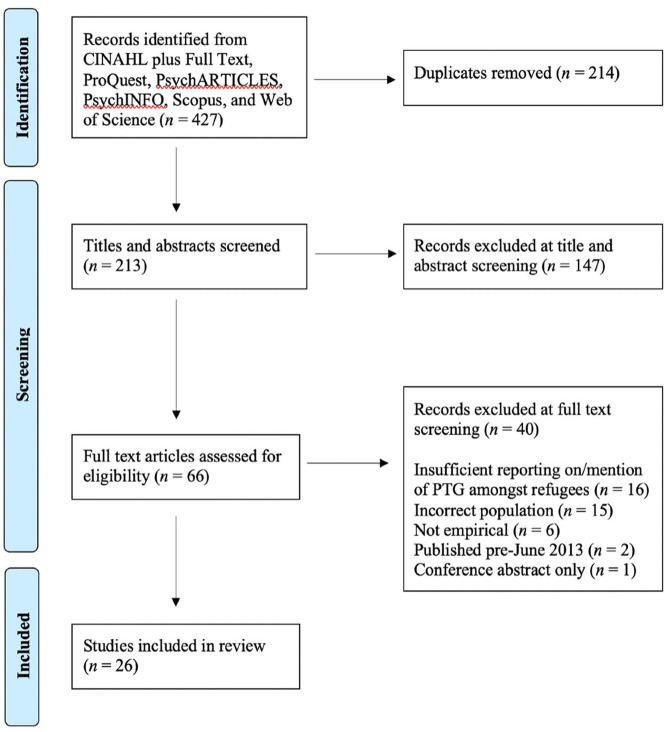
Preferred reporting items for systematic reviews and meta-analyses flowchart.

The PRISMA flowchart (Figure 1) details the process of study inclusion and exclusion. The electronic search of key databases resulted in 427 potentially eligible studies; 213 following de-duplication. A further 147 studies were excluded during title and abstract screening. Of the remaining 66 studies, 40 were excluded during full-text screening for the following reasons: (i) insufficient reporting on/mention of PTG among refugees (*n* = 16), (ii) incorrect population (*n* = 15), (iii) not empirical (*n* = 6), (iv) published pre-June 2013 (*n* = 2), and (v) conference abstract only (*n* = 1). In all, 26 studies met the eligibility criteria and were included in the data extraction and synthesis of findings. A decision was made to include Rizkalla and Segal (2018) as all except one participant were aged >19 years. Two sets of articles reporting on the same research (Study 1: Acquaye, 2017 and Acquaye et al., 2018; Study 2: Alsubaie et al., 2021 and Dolezal et al., 2021) were included in the review.

### Study Characteristics and Quality

As shown in Table 2, studies included in this review used qualitative (*n* = 10), quantitative (*n* = 13), and mixed method (*n* = 3) approaches. Most studies employed a cross-sectional design (*n* = 24), with only two studies (Hijazi et al., 2014; Paloma et al., 2019) using a longitudinal design to explore changes in PTG following intervention. Qualitative studies used interviews (*n* = 8) and focus groups (*n* = 1) (or both; *n* = 1) to explore PTG experiences. All except one quantitative study used the PTGI; Prasetya et al. (2020) did not specify the method of data collection used. The PTGI total mean score was reported in 11 studies. In all, 13 studies used the full 21-item version, including three studies that used the PTGI-Arabic version (Acar et al., 2021; Cengiz et al., 2019; Ersahin, 2020), and one study that used the PTGI-Spanish version (Paloma et al., 2019). Two studies (Alsubaie et al., 2021; Dolezal et al., 2021) used the shortened 10-item version (PTGI-SF). The PTGI-SF displays high internal reliability and correlations with the 21-item tool and can therefore be substituted for the PTGI “with little loss of information” (Cann et al., 2010a, p. 127).

Sample sizes across the included studies ranged from 5 to 805. Two studies included only women (Abraham et al., 2018; Maung et al., 2021) and five included only Muslim participants. In all, 12 studies did not state the religion of participants, and 19 studies explored PTG in refugees from a specific country of origin. Only three studies documented the ethnicity of participants (Ersahin, 2020; Maung et al., 2021; Shakespeare-Finch et al., 2014), two of which explored related experiences of growth. In all, 21 studies explored PTG among refugees or asylum seekers; two studies focused on former refugees (Acquaye, 2017; Acquaye et al., 2018); and three focused on internally displaced persons, either specifically or in addition to refugees and asylum seekers (Alsubaie et al., 2021; Dolezal et al., 2021; Prasetya et al., 2020). Time since relocation reported in the studies ranged from less than 1 year to 47 years.

Application of the QuADS tool (Harrison et al., 2021) (see Table 3) saw the scores of included studies range from very low (Prasetya et al., 2020) to very high (Rizkalla & Segal, 2018; Taher & Allan, 2020). Studies with the lowest scores (Özdemir et al., 2021; Prasetya et al., 2020; Simsir & Dilmac, 2021) lacked appropriate sampling, data collection, and recruitment information. The dearth of stakeholder input and consultation in the research design and conduct was consistently low across most studies, with only two studies (Ferriss & Forrest-Bank, 2018; Maung et al., 2021) receiving full scores for this criterion.

**Table 3.** Quality Assessment of Review Articles (QuADS tool).

	Abraham et al. (2018)	Acar et al. (2021)	Acquaye et al. (2018)	Acquaye (2017)	Alsubaie et al. (2021)	Cengiz et al. (2019)	Dolezal et al. (2021)	Ersahin (2020)	Ferriss and Forrest-Bank (2018)
1. Theoretical underpinning to research	1	3	1	2	2	2	2	3	3
2. Statement of research aims	2	3	3	3	2	2	3	3	2
3. Description of setting/population	2	3	3	1	1	3	1	3	1
4. Appropriate study design	3	3	3	3	2	2	1	2	3
5. Appropriate sampling	1	2	2	1	1	2	2	2	3
6. Rationale for choice of measures	2	3	1	1	1	1	0	1	3
7. Appropriateness & description of measures	1	3	3	3	2	3	2	3	2
8. Description of data collection	2	1	3	1	1	1	1	3	2
9. Recruitment data provided	1	0	2	2	3	0	3	3	1
10. Justification of analytic method	2	0	3	2	0	1	1	0	1
11. Appropriate analysis	2	3	2	2	1	1	2	2	3
12. Consideration of stakeholders in design	0	0	0	0	0	0	0	0	3
13. Strengths and limitations discussed	1	2	2	2	2	1	2	0	2
Total	20	26	28	23	18	19	20	25	29
	Hijazi et al. (2014)	Hirad (2017)	Matos et al. (2021)	Maung et al. (2021)	McCormack and Strezov (2021)	Özdemir et al. (2021)	Paloma et al. (2019)	Prasetya et al. (2020)	Rizkalla and Segal (2018)
1. Theoretical underpinning to research	2	3	2	3	2	2	2	1	2
2. Statement of research aims	1	3	3	3	2	3	1	1	3
3. Description of setting/population	3	2	3	3	2	1	3	2	3
4. Appropriate study design	3	2	3	2	3	1	3	1	3
5. Appropriate sampling	3	3	1	1	1	0	1	1	3
6. Rationale for choice of measures	2	2	1	0	2	0	0	0	3
7. Appropriate description of measures	3	3	3	2	1	2	2	0	3
8. Description of data collection	3	3	3	2	3	0	2	1	3
9. Recruitment data provided	3	1	2	1	1	1	1	1	3
10. Justification of analytic method	3	3	1	3	3	0	1	0	2
11. Appropriate analysis	3	3	3	2	3	2	3	0	3
12. Consideration of stakeholders in design	0	0	2	3	0	0	1	0	0
13. Strengths and limitations discussed	2	3	3	3	2	2	3	0	2
Total	31	31	30	28	25	14	23	8	33
		Shakespeare-Finch et al. (2014)	Simsir and Dilmac, (2021)	Simsir et al. (2021)	Taher and Allan (2020)	Taylor et al. (2020)	Umer and Elliot (2021)	Uy and Okubo (2018)	Wen et al. (2020)
1. Theoretical underpinning to research		3	2	3	2	1	3	3	0
2. Statement of research aims		3	3	2	2	2	2	3	3
3. Description of setting/population		1	1	1	3	1	2	1	3
4. Appropriate study design		3	1	2	3	3	3	3	3
5. Appropriate sampling		1	1	1	3	3	2	3	3
6. Rationale for choice of measures		1	0	1	3	2	0	2	2
7. Appropriate description of measures		1	2	2	3	1	3	3	3
8. Description of data collection		2	1	2	3	3	2	3	2
9. Recruitment data provided		1	1	1	3	1	1	1	2
10. Justification of analytic method		3	2	0	3	2	2	3	2
11. Appropriate analysis		3	2	2	3	3	3	3	3
12. Consideration of stakeholders in design		0	0	2	0	0	1	0	0
13. Strengths and limitations discussed		3	1	2	2	2	3	3	3
Total		25	17	21	33	24	27	31	29

## Quantitative Findings

PTGI mean scores across the studies ranged from 28.86 to 89. Four studies found that women had slightly higher PTG than men (Acquaye, 2017; Ersahin, 2020; Özdemir et al., 2021; Wen et al., 2020). However, this finding was only statistically significant in one study (Acquaye, 2017). One study (Dolezal et al., 2021) provided a comparison of PTG among refugees and asylum seekers, noting that refugees had significantly greater PTG (*t*(94) = 2.71, *p* = .008, *d* = .61).

### PTG and PTSD

Five studies explored the relationship between PTSD and PTG, with all finding that these conditions can co-occur. Examining this co-occurrence, two studies found a positive relationship between PTSD and PTG (*p* < .05; Cengiz et al., 2019; *r* = .167, *p* < .001; Ersahin, 2020), one study identified a curvilinear relationship, with highest PTG found among those with moderate PTSD (Wen et al., 2020), and one study identified no significant relationship between the two constructs (Rizkalla & Segal, 2018).

### Resettlement Experiences

Four studies explored the impact of economic factors and employment on PTG. Rizkalla and Segal (2018) identified that economic factors and employment are salient for PTG, with higher income being the most important factor for enhanced growth. Prasetya et al. (2020) reported that PTG was higher among refugees who were employed, compared to those who were unemployed. In contrast, Özdemir et al. (2021) and Wen et al. (2020) identified no significant association between employment and PTG.

One study (Wen et al., 2020) identified that PTG varied with education levels (*F*(4759) = 4.63, *p* = 0.001), with highest PTG found among those with 12+ years of education, and lowest among those with no formal education.

One study (Acar et al., 2021) found that post-migration stressors were positively associated with growth. Acar et al. (2021) suggested that post-migration difficulties may lead to cumulative stressors, which can result in higher PTG due to a dose–response relationship (i.e., whereby higher stress levels result in increased growth). The authors (Acar et al. 2021) suggest the relationship between post-migration stressors and PTG may result from the use of coping strategies. Similarly, Alsubaie et al. (2021) found that higher levels of discrimination may increase PTG, suggesting that discrimination facilitates religious coping, which, in turn, enhances PTG.

Despite significant variation in resettlement times across studies, only one study assessed the impact of resettlement duration on PTG (Özdemir et al., 2021) finding no significant association.

### Coping Strategies

Religious commitment (the degree to which religious values, beliefs, and practices are adhered to; Worthington et al., 2003) and religious coping (the use of religious beliefs or practices to manage difficult life experiences; Aflakseir & Mahdiyar, 2016) relate to the spiritual domain of PTG and were found to facilitate growth in 13 studies. Three of these studies included only Muslim participants (Alsubaie et al., 2021; Ferriss & Forrest-Bank, 2018; Simsir et al., 2021), two included Muslim and Christian (Acquaye et al., 2018; Maung et al., 2021), and one included Buddhist participants (Uy & Okubo, 2018). Of the remaining seven studies, three included participants from multiple religions (Ersahin, 2020; Hirad, 2017; Shakespeare-Finch et al., 2014), and four did not state the religion of participants (Abraham et al., 2018; Acquaye 2017; Taher & Allan, 2020; Taylor et al., 2020). Religious commitment was identified as a significant predictor of PTG (*r* = .33, *p* = .002; Acquaye et al., 2018), with strength of religiosity strongly relating to overall PTG (*r* = .41, *p* < .001) (Ersahin, 2020). Alsubaie et al. (2021) found that Muslim religious coping also predicted increased PTG (*b* = .62, *p* < .001).

Two studies found that problem-focused coping (an attempt to manage a problem causing stress; Lazarus & Folkman, 1984) is a strong predictor of PTG (Acar et al., 2021; Ersahin, 2020). Emotion-focused coping (an attempt to reduce negative emotions associated with stress; Lazarus & Folkman, 1984) was also found to predict high levels of PTG (Acar et al., 2021). Prasetya et al. (2020) also assessed emotion- and problem-focused coping, finding that the former resulted in lower PTG. This study had a very low-quality assessment score, so caution is warranted when interpreting this result.

Two studies identified dispositional optimism (the tendency to expect good outcomes; Scheier & Carver 2018) and hope, as coping strategies that resulted in higher levels of PTG among refugees (Acquaye, 2017; Umer & Elliot, 2021). Umer and Elliot (2021) found that refugees with high PTG scores looked to the future and set long-term goals focused on work, study, and family, while participants with low PTG scores found it difficult to move forward from the past, and set short-term goals focused on individual survival and safety. These findings link to the PTG domain of “new possibilities.” One study identified a positive correlation between PTG and resilience, with regression analysis showing that resilience promoted growth among refugee participants (Cengiz et al., 2019).

### Trauma Type and PTG

Two studies assessed whether certain types of trauma affect PTG. Özdemir et al. (2021) found no significant relationship between the type of trauma and PTG. In contrast, Acar et al. (2021) identified that the exposure to war and life-threatening events was significantly correlated with the PTG domains of “personal strength” and “spiritual change” (*r* = 0.13, *p* < .001; and *r* = 0.19, *p* < .001, respectively). This study also found that natural disasters and accidents were significantly and positively correlated with all five domains of PTG (*r* = .13 to .19, *p* < .001).

### Interventions to Facilitate PTG

Two studies tested the effects of an intervention to facilitate and promote PTG (Hijazi et al., 2014; Paloma et al., 2019). Hijazi et al. (2014) measured the effects of brief narrative exposure therapy (NET) on PTG and PTSD. Findings revealed that PTG significantly increased following three sessions of brief NET, with the effects lasting up to 4 months (*ES* = 0.83, *p* *<* .001; Hijazi et al., 2014).

Paloma et al. (2019) used a community-based pilot intervention focused on promoting PTG among refugees. This intervention involved two phases; (1) training refugees to become peer mentors and (2) holding cultural peer-support groups led by peer mentors. Pre- and post-test analysis of PTGI data revealed a significant increase in overall PTG following the intervention, (*t*(26) = 3.68, *p* = .001), with significant increases in all domains except “spiritual change.”

## Qualitative Findings

Four themes relating to refugee PTG experiences were developed through the thematic synthesis of qualitative studies, anchored with insightful quotes to foreground participants’ voices: (1) strength from religion—“the beginning and end is all up to God”; (2) comparison-based thinking— “there is no gun behind me here”; (3) survivor’s mission to help others—“I cannot let you struggle”; and (4) the importance of storytelling—“through the narratives of other people, you get closer to your own.”

### Strength from Religion—“the beginning and end is all up to God”

In many studies, religious commitment facilitated meaning making and acceptance of past suffering and traumatic events, enabling participants to regain a sense of power following events that were tragically out of their control: “God wrote for me to be here, and I accept it” (Hirad, 2017, p. 34). Activities such as prayer, and reading or reciting a religious text enabled refugees to manage existing trauma and navigate future hardship, providing a source of strength, comfort, and relief (Hirad, 2017). Some participants also reported an enhanced ability to cope with the injustices of war through a belief that “innocent people will be redeemed, and evil ones will be punished” (Taher & Allan, 2020, p. 342). While most participants reported feeling closer to their faith following refugee trauma (PTG domain of “spiritual change”), some refugees’ relationship with religion shifted considerably after trauma, ranging from a total loss of faith to a reaffirmation of faith (McCormack & Strezov, 2021). Some participants conveyed a feeling of abandonment and betrayal from God (Matos et al., 2021), particularly when the trauma experienced was associated with religion (e.g., religious terrorism; Hirad, 2017).

### Comparison-Based Thinking–“there is no gun behind me here”

Throughout the included studies, many refugees engaged in comparison-based thinking to support positive coping, reflecting on the ways in which their freedom (Ferriss & Forrest-Bank, 2018; Simsir et al., 2021), opportunities (Matos et al., 2021; Simsir et al., 2021), living conditions (Shakespeare-Finch et al., 2014), and sense of safety (Taher & Allan, 2020) had improved since fleeing their homelands. These comparisons extended to the hardship experienced by those less fortunate: “It’s winter, it’s cold out, and I think about those in Afghanistan. . .some of them don’t have a home, a roof over their heads” (Hirad, 2017, p. 29). While upsetting at times (Hirad, 2017), this comparison of circumstances often resulted in increased feelings of gratitude among refugees (PTG domain of “appreciation of life”). Comparison-based thinking also enhanced participants’ confidence in managing future hardships, through recognition of their capacity to survive previous traumatic events (Hirad, 2017; Maung et al., 2021; Uy & Okubo, 2018): “if that didn’t break me, I’ve nothing more to break me” (Taylor et al., 2020, p. 23-24).

### Survivor’s Mission to Help Others—“I cannot let you struggle”

An increase in empathy, compassion, and drive to help others was frequently reported by participants (PTG domain of “relating to others”). Many refugees expressed a sense of responsibility and life purpose to empower and serve the community, particularly those who are suffering (Matos et al., 2021; Uy & Okubo, 2018): “If I am in a free country, then I would be able to help those people, and so that is the point of why I must go on” (Shakespeare-Finch et al., 2014, p. 321). The process of giving back was a common coping strategy among participants; by helping others in need, participants felt happier, less stressed, and less judgmental of others (Maung et al., 2021; Taher & Allan, 2020). The desire to give back also motivated participants to set future-oriented goals, such as a career in the “helping professions” (Shakespeare-Finch et al., 2014; Taylor et al., 2020).

### The Importance of Storytelling—“through the narratives of other people, you get closer to your own”

Many participants reported that being heard and listening to the stories of other refugees strengthened their sense of belonging and social connection: “we share information and our experiences. That is the highlight of my week, coming together with my community” (Hirad, 2017, p. 37; Uy & Okubo, 2018). Through storytelling, refugees established a sense of community (Hirad, 2017), forming local connections through their shared lived experiences (PTG domain of “relating to others”). Listening to others also enabled refugees to reflect on their own experiences (Uy & Okubo, 2018), supporting deeper understanding and personal identification with those facing similar struggles (McCormack & Strezov, 2021; Simsir et al., 2021). For many, this led to a realization that they are not alone in their suffering (Paloma et al., 2019), and a feeling of “empowerment” rather than “sadness” and “anger” (Uy & Okubo, 2018, p. 54). Participants also spoke of the transformative and healing power of storytelling: “after I published my book, I did not have any more nightmares” (Uy & Okubo, 2018, p. 54).

## Discussion

This review synthesized the recent empirical literature on PTG among refugee populations, including the processes that mediate this phenomenon, and the use of therapy in promoting PTG. Findings are discussed with reference to Tedeschi and Calhoun’s (1996) domains of PTG, highlighting new directions for future research and interventions with refugee populations. A summary of critical findings is presented in Table 4, followed by implications for research in Table 5.

**Table 4.** Summary of Critical Findings.

Focus	Critical Finding
PTSD	The variance in findings relating to PTG and PTSD may reflect the level of trauma experienced by different refugee populations
	The varying stressors associated with immigration status may impact findings relating to PTG and PTSD
	Refugees may also be operating from varying baselines levels of trauma, which may have influenced the findings relating to PTG and PTSD
Gender	PTG was mostly found to be higher among women than men, which may result from higher engagement with ruminative thinking and emotion-focused coping among women
Religion	Religious commitment and coping were found to be important in facilitating PTG
Ethnicity	Only three studies documented the ethnicity of participants, two of which explored related experiences of growth
Immigration status	PTG was significantly higher among refugees compared to asylum seekers, which may result from the different post-migration stressors experienced
Interventions	Interventions that involved storytelling, and a community-based approach demonstrated a significant increase in PTG
PTGI	The majority of studies used the PTGI to measure growth; however, only four studies used cross-culturally validated versions

PTGI = post-traumatic growth inventory; PTSD = post-traumatic stress disorder.

**Table 5.** Implications for Research.

Focus	Implications for Research
PTSD	Future research is needed to examine the unique relationship between PTG and PTSD among different refugee populations
Gender	There is a need for future research to better understand the distinct traumatic experiences of refugee men and women, and the complex ways in which these experiences may facilitate, or hinder, PTG
Religion	Mixed methods approaches may prove useful in expanding and deepening understandings obtained from quantitative measures like the PTGI
Ethnicity	Future research is needed to explore the way ethnicity impacts or shapes the PTG experiences of refugee populations
Immigration status	Future research is needed to explore the different impacts of post-migration stressors on the PTG experiences of refugees and asylum seekers
	Future research should also consider how the distinct experiences of IDPs after forced displacement may impact post-trauma outcomes
	A longitudinal approach may provide important insights into the temporal relationship between immigration status and PTG
Interventions	Future research is needed to design and implement narrative interventions for refugee populations, that support healing and transformation through story
	Researchers are encouraged to employ longitudinal methodologies to explore the efficacy of narrative interventions in facilitating PTG among different refugee populations
PTGI	Future research may benefit from using culturally validated measures of PTG
	Researchers are encouraged to explore qualitative accounts to extend existing measures of PTG

IDPs = internally displaced people; PTGI = Post-Traumatic Growth Inventory; PTSD = post-traumatic stress disorder.

This review identified mixed findings in relation to PTG and PTSD; with two studies identifying a positive association (Cengiz et al., 2019; Ersahin, 2020), one study identifying a curvilinear relationship with highest PTG found among those with moderate PTSD (Wen et al., 2020), and one finding no significant relationship between the two constructs (Rizkalla & Segal, 2018). Sims and Pooley (2017) too found the relationship between PTG and PTSD differed between studies, suggesting this variance may reflect the level of trauma experienced by different refugee populations. The varying stressors associated with immigration status may also impact findings relating to PTG and PTSD, particularly among IDPs who can experience difficulty receiving humanitarian assistance (United Nations High Commissioner for Refugees, n.d.-b). Furthermore, it is important to consider that refugees may be operating from varying baselines levels of trauma, which may have influenced the findings relating to PTG and PTSD. Cann et al. (2010b) found that those who have had their core beliefs disrupted by trauma experienced higher levels of PTG than those whose core beliefs were not disrupted. One reason for this is that the disruption of core beliefs facilitates meaning-making processes, which ultimately enhances PTG (LoSavio et al., 2011). These findings highlight the complex and wide-ranging psychological experiences people can have in response to trauma (Papadopoulos, 2007) while also emphasizing the need for future research to examine the unique relationship between PTG and PTSD among different refugee populations.

Four studies in this review compared PTGI scores across genders, identifying higher PTG among women. PTG was also higher among women in Sims and Pooley’s (2017) review (Hussain & Bhushan, 2011; Jayawickreme, 2010), although one study found no significant difference (Powell et al., 2003). The finding that refugee women experience higher levels of PTG is supported by the broader literature, with Vishnevsky et al. (2010) proposing that women are more likely than men to engage in behaviors that facilitate PTG, such as ruminative thinking and emotion-focused coping. However, findings from the broader literature also suggest that refugee women experience unique gender-related trauma and adjustment challenges that worsen their physical and mental anguish (Kheirallah et al., 2022), with Vallejo-Martín et al. (2021) highlighting the unmet need for psychosocial health care in this population. Future research is needed to investigate the distinct traumatic experiences of refugee men and women, and the complex ways in which these experiences may facilitate, or hinder, PTG.

Quantitative studies in this review found that religious commitment and coping facilitate PTG among refugees. Qualitative studies provided further insight into religious commitment and coping as a source of comfort and strength for refugee participants (Hirad, 2017; Matos et al., 2021; McCormack & Strezov, 2021; Taher & Allan, 2020). However, qualitative studies also identified negative impacts of religious commitment and coping, with some participants reporting feeling abandoned and betrayed by God (Matos et al., 2021). At times, these feelings resulted in the complete loss of faith (McCormack & Strezov, 2021). It is possible that the narrative methodologies used in these studies may have facilitated a more nuanced exploration of the relationship between PTG and religion with participants, suggesting that mixed-methods approaches may prove useful in expanding and deepening understandings obtained from quantitative measures like the PTGI.

Only three studies in this review documented the ethnicity of participants, two of which explored related experiences of growth. Sims and Pooley’s (2017) review provided no discussion regarding the impact of ethnicity on refugee PTG experiences. These findings are concerning in light of broader evidence that minority ethnic groups are more likely to experience PTG than others, with Helgeson et al. (2006) suggesting that the higher prevalence of adversity experienced by these groups may increase their propensity for “deriving something good from the bad” (p. 811). Stanton et al. (2006) too found that PTG was higher among Latino people compared to Caucasian, proposing that this may stem from higher engagement with religious coping among Latino people. These findings call for future research exploring the way ethnicity impacts or shapes the PTG experiences of refugee populations.

An emerging finding from this review, not identified by Sims and Pooley (2017), was that PTG was significantly higher among refugees than asylum seekers (Dolezal et al., 2021). This might be due to the unique resettlement issues faced by asylum seekers, such as immigration detention and prolonged asylum application processes; both known to negatively affect mental health outcomes (Masocha & Simpson, 2012). Masocha and Simpson (2012) critiqued the tendency of studies to “lump together” refugees and asylum seekers as a homogeneous group. Future research should, thus, explore the different impacts of post-migration stressors on the PTG experiences of refugees and asylum seekers, to ensure research and interventions capture the voices of, and are responsive to, the needs of these distinct groups. Future research should also consider how the distinct experiences of IDPs after forced displacement may impact post-trauma outcomes. Furthermore, longitudinal research would provide important insights into the temporal relationship between immigration status and PTG.

Both intervention studies identified in this review recorded a significant increase in PTG following intervention. Sims and Pooley (2017) too identified a significant increase in PTG following: (1) a Companion Recovery Program (Gregory & Prana, 2013); (2) brief NET (Hijazi, 2012); and (3) a life story interview and artistic inquiry (Mattoon, 2010). Both reviews revealed the effectiveness of shared narratives in fostering PTG in refugee populations. This highlights an encouraging opportunity for future research to design narrative interventions that support individual healing and promote “social transformations” through story (Uy & Okubo, 2018, p. 57). This is particularly important in light of the finding that some refugee participants had never shared their stories with others (Mattoon, 2010). Future research may also benefit from employing longitudinal methodologies to explore the efficacy of narrative interventions in facilitating PTG among different refugee populations.

The majority of studies in this review used the PTGI to measure growth; however, only four studies used cross-culturally validated versions (Acar et al., 2021; Cengiz et al., 2019; Ersahin, 2020; Paloma et al., 2019). Kashyap and Hussain (2018) caution that the PTGI was developed in a western culture, and originally standardized on a sample of American students. As western societies prioritize individual needs over collective needs (Khatib et al., 2021), the PTGI may, thus, overlook or misinterpret the collectivist values of non-western cultures (Buse et al., 2013). To address these limitations and achieve a more thorough and accurate understanding of diverse PTG experiences, future research with refugee populations may benefit from using culturally validated measures of PTG. In addition to these measures, researchers are encouraged to explore the potential for qualitative accounts to extend existing PTG measures.

## Limitations

This review has several limitations that are worth highlighting. First, the exclusion of studies published in languages other than English may have resulted in a biased sample. Second, to ensure the inclusion of all relevant literature, a decision was made to include studies regardless of time since resettlement. Thus, this review was not able to offer an in-depth exploration of how PTG outcomes may be impacted by the length of resettlement. Finally, the exclusion of studies relating to children and young people from this review meant that not all refugee experiences are accounted for. Considering the unique PTG experiences of children and young people (Kilmer et al., 2014), there is a need for future reviews to include, or focus on, this population.

## Conclusion

This updated review synthesized the recent literature on PTG among refugee populations, including the processes that mediate this phenomenon, and the use of therapy in promoting PTG. The 26 included studies offer important insights into the strengths that can be gained from an individual’s response to refugee trauma. Findings highlight a crucial need for future research to recognize the nuances and complexities of PTG experiences among distinct refugee populations. Qualitative research has an important contribution to make in extending quantitative investigations of PTG among refugees; as an analytical method for in-depth and nuanced exploration into this complex phenomenon, and a foundational ethos of storytelling about lived experiences of transformation and growth.
